# CGI-58/ABHD5 is phosphorylated on Ser239 by protein kinase A: control of subcellular localization[S]

**DOI:** 10.1194/jlr.M055004

**Published:** 2015-01

**Authors:** Anita Sahu-Osen, Gabriela Montero-Moran, Matthias Schittmayer, Katarina Fritz, Anna Dinh, Yu-Fang Chang, Derek McMahon, Andras Boeszoermenyi, Irina Cornaciu, Deanna Russell, Monika Oberer, George M. Carman, Ruth Birner-Gruenberger, Dawn L. Brasaemle

**Affiliations:** * Research Unit Functional Proteomics and Metabolic Pathways, Institute of Pathology, Medical University of Graz, Graz, Austria A-8036, and Omics Center Graz, BioTechMed-Graz, Graz, Austria A-8010; † Rutgers Center for Lipid Research, Rutgers, The State University of New Jersey, New Brunswick, NJ 08901; § Departments of Nutritional Sciences Rutgers, The State University of New Jersey, New Brunswick, NJ 08901; ** Food Science, Rutgers, The State University of New Jersey, New Brunswick, NJ 08901; †† Institute of Molecular Biosciences, University of Graz, Graz, Austria A-8010

**Keywords:** adipocytes, adipose tissue, adipose triglyceride lipase, Chanarin Dorfman syndrome, lipase, lipid droplets, lipolysis, perilipin

## Abstract

CGI-58/ABHD5 coactivates adipose triglyceride lipase (ATGL). In adipocytes, CGI-58 binds to perilipin 1A on lipid droplets under basal conditions, preventing interaction with ATGL. Upon activation of protein kinase A (PKA), perilipin 1A is phosphorylated and CGI-58 rapidly disperses into the cytoplasm, enabling lipase coactivation. Because the amino acid sequence of murine CGI-58 has a predicted PKA consensus sequence of RKYS^239^S^240^, we hypothesized that phosphorylation of CGI-58 is involved in this process. We show that Ser239 of murine CGI-58 is a substrate for PKA using phosphoamino acid analysis, MS, and immuno­blotting approaches to study phosphorylation of recombinant CGI-58 and endogenous CGI-58 of adipose tissue. Phosphorylation of CGI-58 neither increased nor impaired coactivation of ATGL in vitro. Moreover, Ser239 was not required for CGI-58 function to increase triacylglycerol turnover in human neutral lipid storage disorder fibroblasts that lack endogenous CGI-58. Both CGI-58 and S239A/S240A-mutated CGI-58 localized to perilipin 1A-coated lipid droplets in cells. When PKA was activated, WT CGI-58 dispersed into the cytoplasm, whereas substantial S239A/S240A-mutated CGI-58 remained on lipid droplets. Perilipin phosphorylation also contributed to CGI-58 dispersion. PKA-mediated phosphorylation of CGI-58 is required for dispersion of CGI-58 from perilipin 1A-coated lipid droplets, thereby increasing CGI-58 availability for ATGL coactivation.

The control of lipolysis in adipocytes of vertebrates is a carefully orchestrated process. Lipolysis, or the hydrolysis of stored triacylglycerols, releases fatty acids, monoacylg­lycerols, and diacylglycerols that serve as substrates for energy production and the synthesis of phospholipids used for membrane synthesis and repair. The enzymes that catalyze triacylglycerol hydrolysis in adipocytes include adipose triglyceride lipase (ATGL), hormone-sensitive lipase, and monoacylglycerol lipase, to cleave the first, second, and third ester bonds, respectively (1–4). Adipose lipolysis is initiated by a variety of signaling cascades and the ensuing hydrolytic reactions are fine-tuned by an assortment of cytosolic and lipid droplet-associated proteins.

The best-characterized signaling pathway that initiates adipose lipolysis occurs when catecholamines bind to β-adrenergic receptors on adipocyte plasma membranes. Hormone binding triggers a G protein-mediated cascade that activates adenylyl cyclase, increasing levels of cAMP, in turn, activating protein kinase A (PKA). The sub­sequent PKA-mediated phosphorylation of multiple proteins enables lipolysis. These proteins include, but are not limited to, perilipin 1A and hormone-sensitive lipase (2, 5, 6).

Perilipin 1A at the surfaces of lipid droplets is a master regulator of adipose lipolysis. Under basal (fed) conditions, perilipin 1A provides a protective barrier against lipolysis of stored triacylglycerols (7) and binds CGI-58 (also called ABHD5) (8, 9), a coactivator of ATGL (10). When CGI-58 is sequestered on the perilipin scaffold, it cannot interact with or activate ATGL (11). When catecholamines stimulate adipose lipolysis, perilipin 1A is multiply phosphorylated by PKA. The phosphorylation of three N-terminal serine residues of perilipin 1A facilitates the docking of PKA-phosphorylated hormone-sensitive lipase through a protein-protein interaction with the perilipin scaffold (12), thus bringing the lipase to its substrate lipids. The PKA-mediated phosphorylation of two carboxyl-terminal serine residues of perilipin 1A facilitates the release of CGI-58 from perilipin 1A, enabling interaction of CGI-58 with ATGL (11), in turn, activating the lipase (10).

CGI-58 was identified as an important factor in cellular triacylglycerol homeostasis when mutations in CGI-58 were established as the cause of Chanarin Dorfman syndrome (13), a neutral lipid storage disorder (NLSD) characterized by excessive storage of triacylglycerols in many cells and tissues (13–18). CGI-58 was later identified as a coactivator of ATGL (10), thus explaining its role in triacylglycerol turnover; however, the mechanism by which CGI-58 activates ATGL has not yet been elucidated. In this study, we asked whether CGI-58 is a substrate for PKA. We demonstrate that CGI-58 is indeed a substrate for PKA, and show that its phosphorylation is important for the subcellular trafficking of CGI-58 in adipocytes.

## MATERIALS AND METHODS

### Materials

All chemicals were reagent grade. Growth media for cultured cells were obtained from Mediatech, Inc. (Herndon, VA) or Sigma. FBS, isobutylmethylxanthine (IBMX), forskolin, and protease and phosphatase inhibitor cocktails were purchased from Sigma. The DNA purification kit and nickel-nitrilotriacetic acid agarose matrix were purchased from Qiagen. Coomassie Plus protein assay reagent was purchased from Thermo Scientific/Pierce; Bradford and DC protein assays were purchased from Bio-Rad. PfuUltra high-fidelity DNA polymerase was purchased from Stratagene, Inc. (La Jolla, CA). BODIPY 439/503, Alexa Fluor 546 goat-anti rabbit IgG, Alexa Fluor 546 donkey-anti goat IgG, and Alexa Fluor 488 donkey-anti rabbit IgG were purchased from Molecular Probes. Radioactive compounds were purchased from Perkin Elmer Biosciences. Oligonucleotide primers were purchased from Operon BioTechnologies, Inc. (Huntsville, AL). Phospholipids were purchased from Avanti Polar Lipids, Inc. (Alabaster, AL) and Sigma. Bovine heart PKA catalytic subunit and modified trypsin were purchased from Promega. Peptides were synthesized and purified by Bio-Synthesis, Inc. (Lewisville, TX). P81 phosphocellulose paper was from Whatman International Ltd. Hybond-P polyvinylidene difluoride (PVDF) membranes were purchased from Amersham Biosciences. Protein A-Sepharose was from GE Healthcare. All remaining chemicals, scintillation counting supplies, and solvents were purchased from Fisher Scientific or Sigma.

### Expression and purification of recombinant mouse CGI-58, S239A-, S240A-, S239A/S240A-, S239D-, and S239E-mutated CGI-58

Preparation of mouse CGI-58 cDNA subcloned into the pET-28a(+) bacterial expression vector was described previously (19); recombinant CGI-58 included two tandem N-terminal 6-histidine fusion sequences. The cDNA for the S239A-, S240A-, and S239A/S240A-mutated variants of CGI-58 were prepared using PCR to produce two overlapping fragments from the template 12-His-CGI-58 cDNA; fragments were then joined by PCR for subcloning into the pET-28a(+) vector. The mutagenic oligonucleotide primers used to prepare the cDNA for the S239A-mutated variant of CGI-58 were: forward (5′-AAGTACGCGTCTATGTTTGAAGATGACAC-3′), and reverse (5′-ACATAGACGCGTACTTCCGCTTGAAATCAGG-3′) primers. Mutagenic oligonucleotide primers used to prepare the cDNA for the S240A-mutated variant of CGI-58 were: forward (5′-AAGTACTCCGCAATGTTTGAAGATGACACGG-3′) and reverse (5′-AAACATGCGGAGTACTT­CCGCTTGAATCAGG-3′) primers. The cDNA for the S239A/SS240A-mutated variant of CGI-58 was similarly prepared. All mutations were confirmed by DNA sequencing. The 12-His-tagged WT and mutated recombinant S239A, S240A, and S239A/S240A variants of CGI-58 were expressed and purified from *Escherichia coli* cell extracts with nickel-nitrilotriacetic acid agarose, as described (19). All steps for protein purification were performed at 4°C. Enzyme preparations were stored at −20°C.

Additionally, the coding sequences of mouse CGI-58 and mouse ATGL cDNAs were amplified as described (20) for subcloning into the pProEX HTb vector (Addgene, Cambridge, MA), from which the CGI-58 cDNA was subcloned into the His-pSumo vector (kindly provided by Dr. Christopher D. Lima, Sloan Kettering Institute) with a disrupted BamH1 cleavage site. The forward (5′-CGAAGCAGAGAGCTCGAAAACCTGTATT­TT­CAGG-3′) and reverse (5′-GGAACCCTCGAGTCATCAGTCTACTGTGTGGC-3′) oligonucleotide primers included endonuclease cleavage sites for subcloning and a 5′ tobacco etch virus (TEV) cleavage site from the pProEX HTb vector. cDNAs were amplified with PCR using either the Phusion polymerase kit (New England Biolabs) or the FailSafe™ PCR system (Epicenter Biotechnologies, Madison, WI). The S239D and S239E mutations were introduced into the mouse CGI-58 cDNA using the QuikChange^®^ site-directed mutagenesis kit (Stratagene) with forward (5′-CCTGATTTCAAGCGGAAGTACGACTCTATGTTTGAAGATGACA­C­G-3′) and reverse (5′-CGTGTCATCTTCAAACATAGAGTCGTA­CTTCCGCTTGAAA­TCAGG-3′) mutagenic primers for S239D CGI-58 and forward (5′-CCTGATTTCAAGCGGAAGTACGA­GTCTATGTTTGAAGATGACACG-3′) and reverse (5′-CGT­G­TC­ATCTTCAAACAT­AGACTCGTACTTCCGCTTGAAATCAGG-3′) mutagenic primers for S239E. The mouse ATGL cDNA encoding amino acids 1–288 was subcloned into His-pSumo with 5′ TEV site using forward (5′-GCTATGGATCCATGTTCCCGAGGG-3′) and the reverse (5′-GGCGCTCGAGTCATTTTTCGAACTGCGG­GTGGCTCCAATCCTCCTCT­CCAGC-3′) oligonucleotide primers including endonuclease cleavage sites and a stop codon following the nucleotide sequence for D288, followed by the sequence encoding a Strep-tag^®^ (IBA, Goettingen, Germany). Mutations were confirmed by DNA sequencing (Integrated DNA Technologies, Coralville, IA and Agowa, Berlin, Germany).

His-pSumo-CGI-58, His-pSumo-ATGL 1-288, and the His-pSumo vector encoding 6-His-smt protein were transformed into BL21(DE3) RIPL CodonPlus *E.* *coli* (Agilent Technologies). Expression of proteins was induced with 0.5 mM isopropyl β-D-1-thiogalactopyranoside when cells reached an optical density of 0.6 at 600 nm. Induced His-pSumo-CGI-58 cells were grown for 9–12 h, while His-pSumo-ATGL 1-288 and His-pSumo cells were grown for 4 h at 30°C. Expression of the proteins was confirmed with SDS-PAGE.

*E. coli* expressing recombinant CGI-58 in His-pSumo were disrupted by probe sonication in buffer-1 [20 mM Tris-HCl, 500 mM NaCl, 30 mM imidazole, 0.1% NP-40, 3.5 mM β-mercaptoethanol (pH 7.8)] supplemented with protease inhibitor cocktail (Complete, EDTA-free Tabs-Roche), 750 U benzonase nuclease HC (Novagen), and 1 mg/ml lysozyme. Cell debris was removed by centrifugation at 30,000 *g* for 40 min and soluble recombinant CGI-58 was purified with affinity chromatography using a 5 ml His-Trap FF column (GE Healthcare). The column was washed extensively with buffer-1 and eluted with a linear gradient of 10 column volumes with buffer-2 [20 mM Tris-HCl, 500 mM NaCl, 250 mM imidazole, 10% glycerol, 3.5 mM β-mercaptoethanol (pH 7.8)]. Cleavage with tobacco etch virus protease was performed at room temperature for 4 h and the cleaved CGI-58 was further purified on a Superdex 200 (Sigma-Aldrich) column equilibrated in buffer-3 [20 mM Tris-HCl, 300 mM NaCl, 1 mM DTT, 1 mM EDTA (pH 7.8)]. Residual 6-His-smt was removed by reverse affinity chromatography with a 5 ml His-Trap FF column equilibrated in buffer-3. Recombinant CGI-58 was then dialyzed into the PKA assay buffer.

*E. coli* expressing recombinant ATGL 1-288 and 6-His-smt protein were disrupted by sonication in buffer-4 [50 mM Tris-HCl, 300 mM NaCl, 10% glycerol, 20 mM imidazole, 1 mM benzamidine, 1 mM Tris(2-carboxyethyl)phosphine (TCEP) (pH 7.8)] supplemented with 0.1 mM phenylmethanesulfonylfluoride and protease inhibitor cocktail. Cell debris was removed by centrifugation and soluble protein was loaded onto a 1 ml His-Trap FF column. The column was washed extensively with buffer-4 and the proteins were eluted with a gradient of 12 column volumes of buffer-5 [50 mM Tris-HCl, 300 mM NaCl, 10% glycerol, 500 mM imidazole, 1 mM benzamidine, 1 mM TCEP (pH 7.8)]. Subsequently, ATGL 1-288 was dialyzed into buffer-6 [50 mM Tris-HCl, 300 mM NaCl, 10% glycerol, 1 mM EDTA, 1 mM TCEP (pH 7.8)] and used directly in the triacylglycerol hydrolase activity assay.

### Phosphorylation of recombinant mouse CGI-58 and synthetic peptides with PKA

Peptides of 10 amino acids surrounding and including the RKYSS sequence, with peptides containing RKYSS, RKYSA, RKYAS, and RKYAA, were tested for phosphorylation by PKA. Additionally, the PKA-mediated phosphorylation of comparable variants of purified recombinant CGI-58 was examined. Both synthetic peptides and variants of purified recombinant CGI-58 (∼0.5–0.8 μg) were incubated with the indicated concentrations of PKA and 50 μM [γ^32^P]ATP (∼230,000–470,000 cpm/nmol) in 10 mM MgCl_2_, 60 mM DTT, 50 mM Tris-HCl (pH 8.0); reactions were incubated for 10 min at 30°C. Reactions were stopped by the addition of 4× Laemmli’s sample buffer (21), followed by SDS-PAGE, immunoblot analysis, and autoradiography, or by spotting the reaction mixtures onto Whatman P81 phosphocellulose filters, followed by repeated treatments of the filters with 75 mM phosphoric acid and drying. All phosphorylation reactions were performed in triplicate. A unit of PKA was defined as the amount of enzyme that catalyzed the formation of 1 nmol of product/min. For some experiments, the PKA phosphorylation reaction was performed with nonradioactive ATP.

### Phosphoamino acid analysis

For phosphoamino acid analysis, a portion of a PVDF membrane containing ^32^P-labeled recombinant CGI-58 was subjected to acid hydrolysis with 6 N HCl (22). Hydrolysates were dried under vacuum and applied to cellulose TLC plates (EM Science) with standard phosphoamino acids, phosphoserine, phosphothreonine, and phosphotyrosine. Amino acids were separated by 2D electrophoresis using formic acid:acetic acid:water (50:156:1,794, v/v) in the first dimension and acetic acid:pyridine:water (100:10:1,890, v/v) in the second dimension (23). Following electrophoretic separation, the TLC plates were dried and subjected to phosphorimaging analysis with a Molecular Dynamics Storm phosphorimager. Standard phosphoamino acids were visualized by spraying the plate with 0.25% ninhydrin in acetone.

### MS

Purified recombinant CGI-58 was phosphorylated in vitro by incubation with PKA and nonradioactive ATP. Phosphorylated residues of CGI-58 were identified by LC-MS/MS after reduction, alkylation, and enzymatic digestion with AspN, modified trypsin (Promega), or chymotrypsin (Roche). Digests of 0.5 μg CGI-58 were acidified with 0.5% trifluoroacetic acid and separated by nano-HPLC (Dionex Ultimate 3000) equipped with a μ-precolumn (C18, 5 μm, 100 Å, 5 × 0.3 mm) and an Acclaim PepMap RSLC nanocolumn (C18, 2 μm, 100 Å, 150 × 0.075 mm) (all Thermo Fisher Scientific, Vienna, Austria). Samples were concentrated on the enrichment column for 2 min at a flow rate of 20 μl/min with 0.5% trifluoroacetic acid as an isocratic solvent. Separation was carried out on the nanocolumn at a flow rate of 300 nl/min using the following gradients, where solvent A is 0.3% formic acid in water and solvent B is a mixture of 80% acetonitrile in water containing 0.3% formic acid: 0–2 min 4% B, 2–35 min 4–28% B, 35–47 min 28–50% B, 47–48 min 50–95% B, 48–58 min 95% B, 58–58.1 min 95–4% B, 58.1–70 min 4% B, or 0–2 min 4% B, 2–180 min 4–28% B, 180–255 min 28–50% B, 255–260 min 50–95% B, 260–279 min 95% B, 279–280 min 95–4% B, 280–300 min 4% B. The sample was ionized in the nanospray source equipped with stainless steel emitters (ES528, Thermo Fisher Scientific). It was analyzed in an Orbitrap Velos Pro™ mass spectrometer (Thermo Fisher Scientific, Waltham, MA) operated in positive ion mode, applying alternating full scan MS (*m/z* 400–2,000) in the ion cyclotron and MS/MS by high energy collision-induced dissociation of the 10 most intense peaks in the Orbitrap with dynamic exclusion enabled or neutral loss MS^3^ scanning for the loss of phosphate in the ion trap.

The LC-MS/MS data were analyzed by searching the mammalian SwissProt public database with Proteome Discoverer 1.4 (Thermo Fisher Scientific) and Mascot 2.4 (MatrixScience, London, UK). Carbamidomethylation on Cys was entered as a fixed modification. Oxidation on methionine and phosphorylation on serine or threonine was entered as a variable modification. A precursor mass error tolerance of 10 ppm and a product mass error tolerance of 0.7 Da were used. A maximum false discovery rate of 5% using decoy database search and a Mascot ion score cut off of 30 were chosen as identification criteria.

### Measurement of CGI-58 coactivation of ATGL in vitro

Purified recombinant CGI-58 was phosphorylated in vitro by incubation with PKA and nonradioactive ATP. Both phosphorylated and nonphosphorylated proteins were incubated with an Sf9 insect cell lysate expressing recombinant mouse ATGL (24) and a radioactive triacylglycerol substrate emulsified with phospholipids, as described previously (10). Fatty acids were extracted from the reaction mixture with solvents and quantified by scintillation counting (10).

In other experiments, recombinant CGI-58, S239D CGI-58, and S239E CGI-58 were expressed in *E. coli*, followed by disruption of the cells by sonication and removal of cell debris by centrifugation for 10 min at 2,700 *g* and determination of the protein concentration of the extracts by Bradford assay. Twenty-six micrograms of the supernatants with variants of recombinant CGI-58 were used in triacylglycerol hydrolase assays with 8 μg of partially purified recombinant ATGL 1-288, as described (25).

### Generation of recombinant adenovirus

Adenoviral expression vectors driving the expression of mouse CGI-58 and β-galactosidase were described previously (19). Adenoviral expression vectors driving expression of the S239A/S240A-mutated variant of CGI-58 and perilipin 1A were prepared by ligating the cDNA for S239A/S240A-mutated CGI-58, mouse perilipin 1A, or mutated perilipin 1A lacking serine residues in six consensus sites for PKA phosphorylation into the shuttle vector for the AdEasy XL adenoviral vector system (Stratagene), and then following the manufacturer’s protocols for recombination of the shuttle vector to make adenoviral expression vectors and for assembly of virions in cultured AD293 cells. The mutated perilipin 1A cDNA encoded alanine substitutions for serine residues at positions 81, 222, 276, 433, and 492 to prevent phosphorylation by PKA, and a glutamate substitution for serine 517 to permit targeting of the mutated perilipin 1A to lipid droplets (26). Adenoviral preparations were purified over cesium chloride gradients.

### Cell culture and adenoviral transduction

Normal human skin fibroblasts (WS1) were obtained from American Type Culture Collection (Manassas, VA); human skin fibroblasts from an individual with NLSD (27–29) were generously provided by Dr. Rosalind A. Coleman (University of North Carolina, Chapel Hill, NC). Normal and NLSD fibroblasts were cultured as described previously (27–29). NLSD fibroblasts were transduced with purified adenoviral preparations for expression of intact (WT) mouse CGI-58 (WT) or S239A/S240A-mutated CGI-58; cells were collected at various times following transduction for the determination of triacylglycerol levels.

For other experiments, purified adenoviruses were used to transduce Cos-7 cells or NIH3T3CARΔ fibroblasts. Cos-7 cells were cultured in DMEM supplemented with 10% FBS, 100 units/ml penicillin, and 100 μg/ml streptomycin. NIH3T3CARΔ fibroblasts are NIH3T3 fibroblasts stably expressing a truncated version of the Coxsackie and adenovirus receptor (CARΔ) lacking the cytoplasmic signaling domain (30) to increase the uptake of adenoviral vectors. The cells were cultured in DMEM supplemented with 10% calf serum, 100 units/ml penicillin, and 100 μg/ml streptomycin, with 800 μg/ml G418 to maintain selection of cells expressing CARΔ. Cells were transduced with adenoviruses for the expression of either WT mouse perilipin 1A or the mutated variant of mouse perilipin 1A that lacks six serine residues in PKA consensus sequences. At the same time, the cells were also transduced with either WT CGI-58 or S239/S240A CGI-58 and incubated for 48 h before processing the cells for immunofluorescence microscopy.

### Immunofluorescence microscopy

Twenty-four hours after transduction, Cos-7 and NIH3T3CARΔ cells expressing perilipin 1A or mutated perilipin 1A and CGI-58 or S239A/240A-mutated CGI-58 were transferred to glass coverslips. Thirty-six hours after transduction, 200 μM oleic acid complexed to fatty acid-free BSA at a 4:1 molar ratio (7) was added to increase the synthesis and storage of triacylglycerol in lipid droplets. Forty-eight hours after transduction, cells were incubated with 10 μM forskolin and 0.5 mM IBMX in culture medium supplemented with 1% fatty acid-free BSA for 30 min for stimulated conditions, or in culture medium with 1% fatty acid-free BSA for 30 min for basal conditions. Cells were fixed with 4% paraformaldehyde in PBS for 20 min, followed by a PBS wash step, and prepared for microscopy, as described previously (31). Cells expressing perilipin 1A and CGI-58 were probed with goat polyclonal antisera raised against an N-terminal peptide of perilipin 1A (kindly donated by Dr. Constantine Londos, formerly of National Institutes of Health, Bethesda, MD, deceased) and rabbit polyclonal antisera raised against recombinant mouse CGI-58 (8), followed by anti-goat Alexa Fluor 546 and anti-rabbit Alexa Fluor 633. Nuclei were stained with Hoechst 33422 and lipid droplets were stained with BODIPY 493/503 (Life Technologies) (32), each at 0.1 μg/ml in PBS for 10 min. Cells were viewed with either a Nikon Eclipse E800 fluorescence microscope equipped with a Photometrics CoolSNAP EZ digital camera or a LSM510 Meta confocal laser scanning microscope (Zeiss, Oberkochen, Germany) using a 63× oil immersion lens. Images were processed using Zen 2008 and ImageJ software. Cells were manually scored for protein localization patterns by at least two observers blinded to sample identity and automatically scored using the ImageJ JACoP plugin (33) to determine Manders coefficients; more than 50 cells were counted for each determination.

### Measurement of cellular triacylglycerol

Human NLSD fibroblasts were transduced with adenovirus to drive the expression of WT CGI-58 or S239A/S240A-mutated CGI-58. At various times after transduction, cells were harvested and lysed in hypotonic lysis solution (10 mM Tris-HCl, 10 mM NaF, 1 mM EDTA, 10 μg/ml leupeptin, 100 μM 4-(2-aminoethyl)benzenesulfonylfluoride hydrochloride, and 500 μM benzamidine) and homogenized by probe sonication (Branson Sonifier) for 10 s. Lipids were extracted from a portion of samples using isopropanol:hexane:water (80:20:2), and the lipid phase was collected and evaporated. Lipid extracts of corn oil were used as triacylglycerol standards. The cellular triacylglycerol content was determined using an enzymatic assay from Thermo Electron Trace adapted for use with cultured cells (34). Absorbance of samples was determined at 540 nm using a VersaMax microplate reader (Molecular Devices). The triacylglycerol measurements were expressed relative to cellular protein content measured by the Bio-Rad DC Protein Assay.

In one experiment, 6 h after transduction of NLSD fibroblasts with intact or S239A/S240A-mutated CGI-58, 10 μM forskolin and 0.5 mM IBMX were added to increase adenylyl cyclase activity, in turn, activating PKA. Cells were collected at 0, 0.5, 1, and 2 h for the determination of triacylglycerol levels.

### Adipose tissue collection

Animal care and handling were performed in accordance with the standards established by the Austrian Federal Ministry of Science and Research, Division of Genetic Engineering and Animal Experiments (Vienna, Austria) and protocols were approved by an institutional review board. Mice had ad libitum access to food and water under a 12 h light/12 h dark cycle in a temperature-controlled environment. Male C57Bl/6 mice (age 8–12 weeks, body weight 20–30 g) were fed a chow diet (Ssniff^®^, Soest, Germany) and fasted during the daytime for 6 h prior to euthanization. Gonadal white adipose tissues were collected and cut into small pieces. Tissue samples were incubated in low glucose DMEM supplemented with 2% fatty acid-free BSA, with half of the tissue fragments additionally incubated with 10 μM isoproterenol and 0.5 mM IBMX for 20 min at 37°C with gentle shaking. Tissues were then lysed by sonication in ice-cold PBS (pH 7.4), with protease and phosphatase inhibitors. Lysates were centrifuged at 10,000 *g* for 10 min at 4°C. Protein content of supernatants was determined by Bradford Assay.

### Immunoprecipitations

Forskolin-treated and untreated control white adipose tissue lysates and lysates from NIH3T3CARΔ fibroblasts expressing intact or 239A/S240A-mutated CGI-58 were precleared with Protein A-Sepharose beads. CGI-58 antiserum (8) was cross-linked to Protein A-Sepharose using dimethyl pimelimidate. Precleared protein lysates (1 mg protein) were incubated with cross-linked antiserum for 16 h at 4°C; control immunoprecipitations were conducted using control IgG. Immunoprecipitates were collected by centrifugation at 1,000 *g* for 1 min at 4°C. After washing the beads three times with immunoprecipitation buffer [50 mM Tris-HCl (pH 7.4), 300 mM NaCl, 0.1% Triton X-100, 5 mM EDTA, 50 mM NaF, 0.02% NaN_3_, with protease and phosphatase inhibitors], proteins were eluted using hot SDS-PAGE XT (Bio-Rad) sample buffer for 10 min at 90°C. Proteins were separated by electrophoresis on 4–20% gradient gels for SDS-PAGE (Bio-Rad) and transferred to nitrocellulose membranes. Membranes were immunoblotted with anti-CGI-58 (1:50,000) (8) or phospho-S/T-PKA substrate antibody (1:1,000) (#9621; Cell Signaling Technology, Danvers, MA) in 5% (w/v) BSA in TBS with 0.1% Tween-20 overnight at 4°C. Blots were incubated with Clean-Blot IP detection reagents (Thermo Scientific) (1:2,500) in 5% (w/v) nonfat milk in TBS with 0.02% Tween-20 for 2 h at 25°C, followed by detection with SuperSignal West Femto chemiluminescent substrate (Thermo Scientific).

### SDS-PAGE, 2D electrophoresis, and immunoblot analysis

SDS-PAGE, 2D gel electrophoresis, and immunoblotting of proteins on nitrocellulose membranes were performed using standard procedures. For 2D gel electrophoresis, proteins were separated in the first dimension on immobilized nonlinear pH gradient strips (pH 3–10) (Bio-Rad) by a gradual increase in voltage from 200 V to 8,000 V at the rate of 4 V/min (28 kVh in total). The second dimensional separation was performed using 4–12% gradient SDS-PAGE gels (Bio-Rad). Antibodies used for immunoblotting were rabbit polyclonal anti-mouse CGI-58 antiserum (8), rabbit polyclonal anti-β-galactosidase antibody (#ab616; Abcam, Cambridge, MA), rabbit polyclonal anti-calnexin antibody (#SPA-865; StressGen, Victoria, BC, Canada), phospho-S/T-PKA substrate antibody (#9621; Cell Signaling Technology, Danvers, MA), and polyclonal antisera raised against an N-terminal recombinant peptide of perilipin A (35). Proteins were detected using peroxidase-conjugated goat anti-rabbit IgG (Sigma) and enhanced chemiluminescence reagents (Amersham Biosciences or Thermo Scientific).

### Data and statistical analyses

Kinetics data were analyzed according to the Michaelis-Menten equation using the GraphPad Prism kinetic model-fitting program. Data are reported as mean ± standard deviation or standard error and were analyzed using two-way ANOVA and Tukey’s post hoc test with SPSS software. Differences between samples were considered significant at *P* < 0.05.

## RESULTS

### Recombinant CGI-58 is phosphorylated by PKA in vitro

**Fig. 1.**
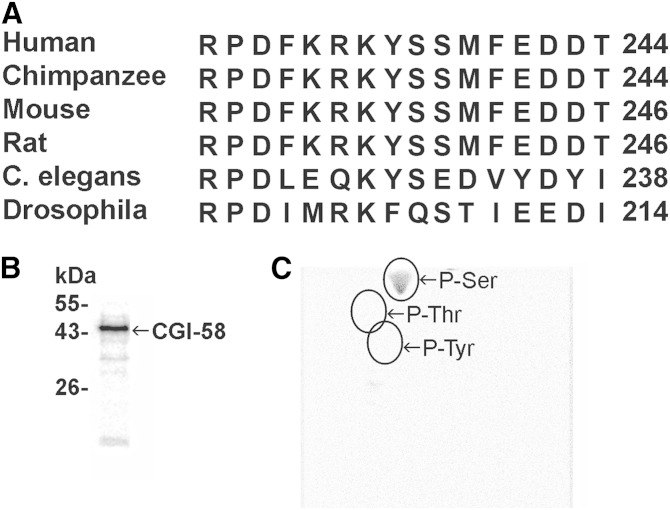
CGI-58 is a substrate for in vitro phosphorylation by PKA. A: Amino acid sequences of a portion of CGI-58 are aligned for human (NP_057090), chimpanzee (XP_516397), mouse (NP_080455), rat (NP_997689), *C. elegans* (hypothetical protein C37H5.2, NP_504299.2), and *D. melanogaster* (CG1882, isoform B, NP_724609.1). The sequences show conservation of the RKYSS consensus site for PKA phosphorylation in vertebrates, but not invertebrates. B: Recombinant CGI-58 (1 μg) was incubated with PKA (0.32 unit/ml) and 50 μM [γ-^32^P]ATP (236,000 cpm/nmol) for 15 min, followed by SDS-PAGE, transfer to PVDF, and phosphorimaging analysis. C: A PVDF membrane slice containing ^32^P-labeled CGI-58 was hydrolyzed with 6 N HCl, followed by 2D electrophoretic analysis. The positions of the standard phosphoamino acids, phosphoserine (P-Ser), phosphothreonine (P-Thr), and phosphotyrosine (P-Tyr), are indicated. The data are representative of two independent experiments.

Analysis of the predicted amino acid sequence of mouse CGI-58 using PROSITE (36) revealed a PKA consensus sequence of RKYSS containing two potential targets for serine phosphorylation, Ser239 and Ser240. This consensus sequence is highly conserved in the amino acid sequences of CGI-58 from vertebrate species, but not in the sequences of CGI-58 orthologs from *Caenorhabditis elegans* or *Drosophila melanogaster* (**Fig. 1A**). To test the hypothesis that CGI-58 is a substrate for PKA, we studied the phosphorylation of recombinant mouse CGI-58 that was purified from *E.* *coli* lysates (19). Recombinant CGI-58 was incubated with mammalian PKA and [γ-^32^P]ATP in vitro; the incorporation of radioactive phosphate into CGI-58 was monitored by phosphorimaging analysis of blots and revealed that recombinant CGI-58 is a substrate for PKA (Fig. 1B). Phosphoamino acid analysis of the ^32^P-labeled protein showed that PKA phosphorylates CGI-58 at a serine residue (Fig. 1C).

**Fig. 2.**
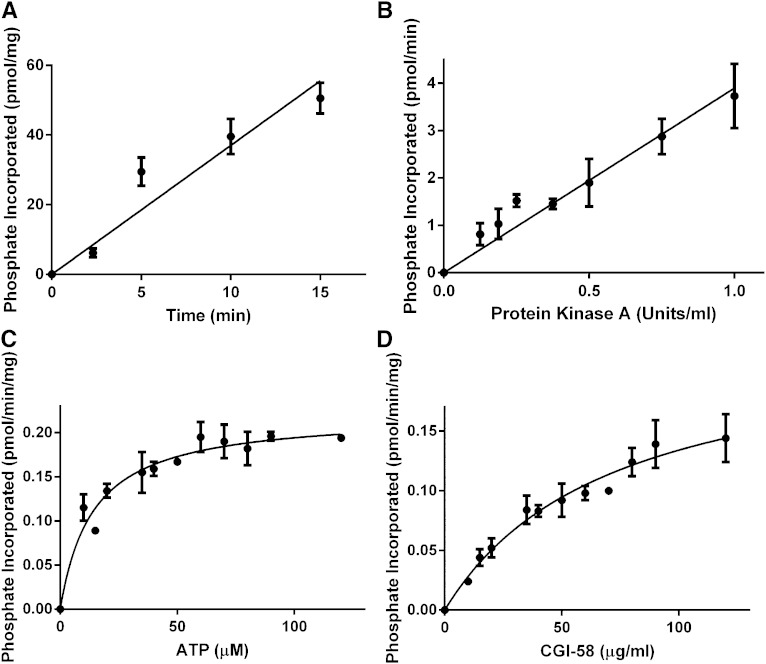
Kinetics of the in vitro phosphorylation of recombinant CGI-58 by PKA. A: Time dependence. Recombinant CGI-58 (1 μg) was incubated with PKA (0.32 unit/ml) and 50 μM [γ-^32^P]ATP for the indicated time intervals. B: Concentration of PKA. Recombinant CGI-58 (1 μg) was incubated with the indicated concentrations of PKA and 50 μM [γ-^32^P]ATP for 10 min. C: Concentration of ATP. Recombinant CGI-58 (1 μg) was incubated with PKA (0.32 unit/ml) and the indicated concentrations of [γ-^32^P]ATP for 10 min. D: Concentration of CGI-58. The indicated concentrations of recombinant CGI-58 were incubated with PKA (0.32 unit/ml) and 50 μM [γ-^32^P]ATP for 10 min. After the phosphorylation reactions, the samples were spotted onto Whatman P81 paper, washed with 75 mM phosphoric acid, and radioactivity determined by scintillation counting. The data for each panel are the mean ± standard deviation of triplicate samples from one representative experiment out of two. When error bars are not visible, they are contained within the symbol.

The kinetics of the in vitro phosphorylation reaction were studied in detail. Phosphorylation of CGI-58 by PKA displayed saturation kinetics and was dependent on time (**Fig. 2A**), concentration of PKA (Fig. 2B), concentration of ATP (Fig. 2C), and concentration of recombinant CGI-58 (Fig. 2D). Analysis of the data using the Michaelis-Menten equation yielded apparent *K_m_* values for ATP and recombinant CGI-58 of 14.0 ± 2.8 μM and 71.5 ± 13.9 μg/ml, respectively.

### PKA phosphorylates CGI-58 at Ser239

The predicted PKA consensus sequence of mouse CGI-58 has potential phosphorylation sites at Ser239 and Ser240 (Fig. 1A). To determine whether these serine residues are substrates for PKA, 10-amino acid peptides containing the intact PKA consensus sequence of RKYSS or comparable peptides with alanine substitutions for these serine residues both individually and in combination (S239A, S240A, S239A/S240A) were synthesized. We examined whether these peptides serve as substrates for PKA in vitro. Both the peptide with the intact RKYSS consensus sequence and the S240A peptide were highly phosphorylated (**Table 1**). In contrast, the S239A peptide was a poor substrate for PKA, showing only 6% of the activity of the peptide with intact RKYSS. The S239A/S240A peptide was not phosphorylated by PKA in vitro. These data indicate that Ser239, in the context of the local amino acid environment, serves as a substrate for PKA in vitro.

**TABLE 1.** PKA-mediated phosphorylation of peptides of CGI-58

Peptide	Sequence	PKA Activity (μmol/min/mg)
S239/S240 (WT)	FKRKYSSMFE	43.1 ± 2.1
S240A	FKRKYSAMFE	45.8 ± 4.7
S239A	FKRKYASMFE	2.54 ± 0.32
S239A/S240A	FKRKYAAMFE	0.04 ± 0.02

Ten-amino acid peptides (0.5 mM) were incubated with PKA (0.32 unit/ml) and 50 μM [γ-^32^P]ATP for 10 min and then spotted onto Whatman P81 phosphocellulose filters, followed by rinses with 75 mM phosphoric acid and drying. Incorporation of phosphate into peptides was determined by scintillation counting. Data are mean ± standard deviation of triplicate reactions.

**Fig. 3.**
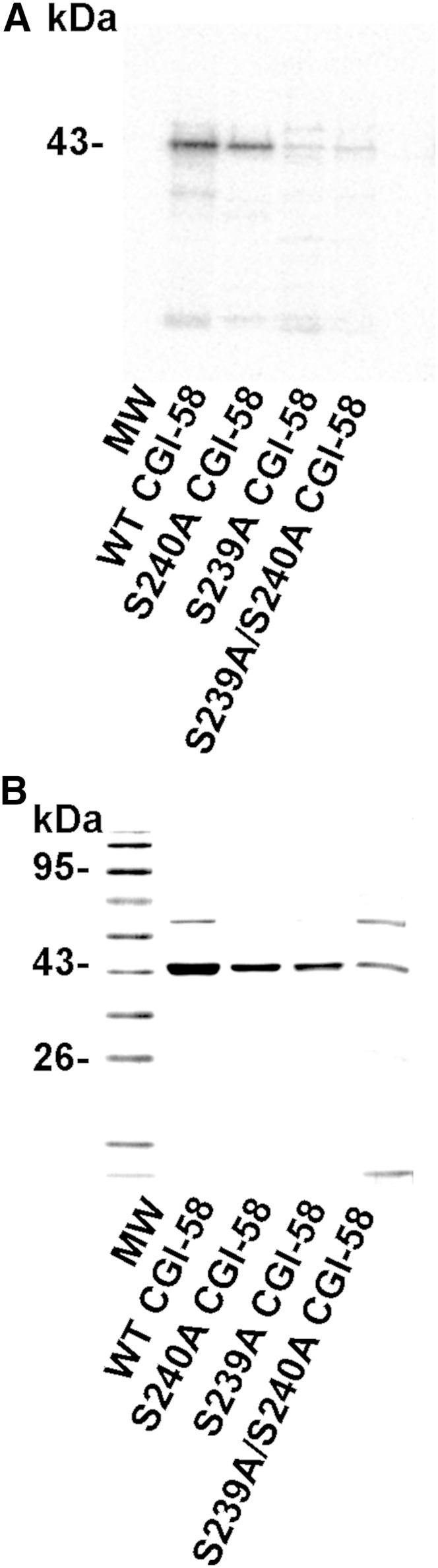
CGI-58 is phosphorylated in vitro by PKA on Ser239. Recombinant CGI-58 (WT) or S240A-, S239A-, or S239A/S240A-mutated CGI-58 (1 μg) were incubated with PKA (0.32 unit/ml) and 50 μM [γ-^32^P]ATP before SDS-PAGE, transfer to PVDF, and phosphorimaging analysis (A). B: Coomassie-stained gel prior to protein transfer.

We then extended these studies to full-length recombinant CGI-58. Recombinant variants of CGI-58 with the intact RKYSS sequence, or comparable mutations (S239A, S240A, S239A/S240A), were expressed and purified from *E. coli* lysates, and then incubated with PKA and [γ-^32^P]ATP in vitro. Autoradiography of SDS-PAGE gels containing the recombinant proteins revealed that the S239A-mutated variant of recombinant CGI-58 is a poor substrate for PKA, whereas CGI-58 with the intact RKYSS sequence, and S240A-mutated CGI-58 are good substrates for PKA (**Fig. 3**). These data suggest that the major site of phosphorylation is Ser239.

MS was used to further study the in vitro phosphorylation of CGI-58. Recombinant mouse CGI-58 was purified from *E. coli* lysates, incubated with PKA and ATP, reduced, alkylated and digested with trypsin, chymotrypsin, or AspN. Digests were then analyzed by LC-MS/MS using different fragmentation and scanning techniques. A search of the mammalian SwissProt database unambiguously identified Ser239 as the major phosphorylation site in CGI-58 peptides derived from three different digests (**Table 2**). The majority of peptides containing Ser239 were phosphorylated.

**TABLE 2.** Phosphorylated peptides of CGI-58 identified by MS

Enzyme	Sequence	*m/z*	Δm (ppm)	Ion Score	Expect Value
Trypsin	KYsSMFEDDTVTEYIYHCNVQTPSGETAFK	1,209.85059	1.09	104	2.9E-009
AspN	DFKRKYsSmFE	767.32617	0.42	32	7.5E-003
Chymotrypsin	KRKYsSMF	571.75867	3.16	30	6.9E-003

Purified recombinant CGI-58 was incubated with PKA in vitro, digested with either trypsin, AspN, or chymotrypsin, and analyzed by LC-MS/MS. Peptides were identified by searching the mammalian SwissProt database with Proteome Discoverer and Mascot software. Modified amino acids are shown in small letters (s designates phosphorylated serine, m designates oxidized methionine). The *m/z* and mass error (Δm) of measured peptides, as well as Mascot ion scores and expected values, are listed.

**Fig. 4.**
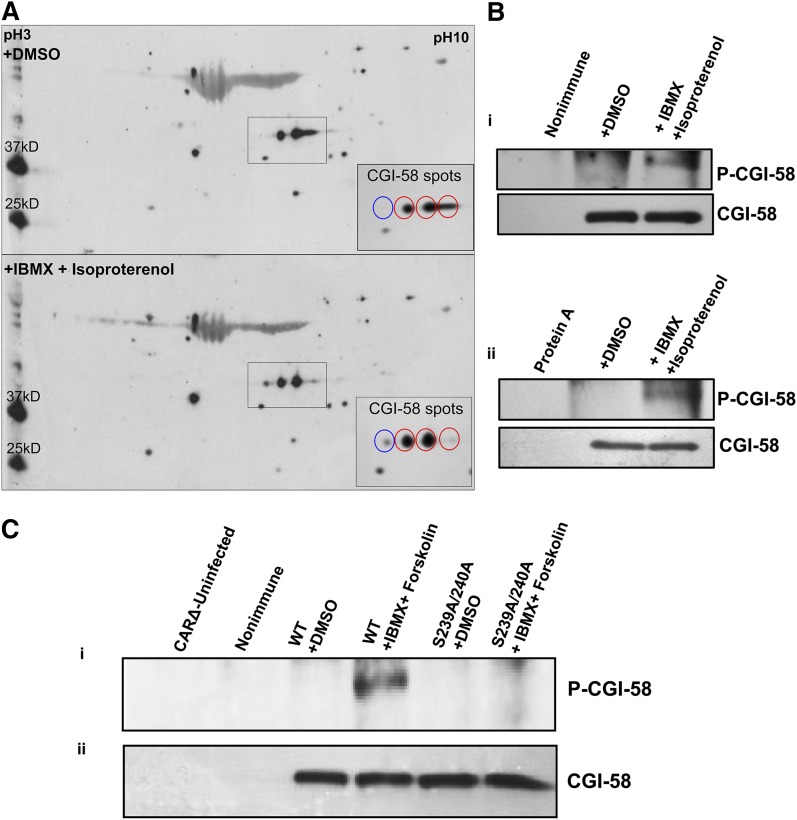
CGI-58 is a substrate for PKA in white adipose tissue and intact cells. White adipose tissue sections from C57B1/6 mice were incubated with 0.5 mM IBMX and 10 μM isoproterenol for 20 min at 37°C (A, B). A: Immunoblot of 2D electrophoresis gel of stimulated and control white adipose tissue lysates probed with CGI-58 antiserum. Inset shows an enlargement of the boxed region. B: Immunoblots of CGI-58 immunoprecipitated from tissue lysates made from stimulated and control white adipose tissue probed with PKA pS/T substrate antibody (P-CGI-58) and CGI-58 antiserum. Control immunoprecipitations with control IgG showed no signal for CGI-58 [lane 1 (i)]; similarly, precipitations conducted without IgG, but with Protein A-Sepharose, showed no signal for CGI-58 [lane 1 (ii)]. C: NIH3T3CARΔ cells expressing intact CGI-58 (WT) or S239A/240A-mutated CGI-58 were incubated with 0.5 mM IBMX and 10 μM forskolin for 20 min at 37°C. Immunoblots show CGI-58 immunoprecipitated from cell lysates made from stimulated and control NIH3T3CARΔ cells expressing WT CGI-58 or S239A/S240A-mutated CGI-58 probed with PKA pS/T substrate antibody (i) and CGI-58 antiserum (ii). Phosphorylated protein was detected only when WT CGI-58 was expressed in cells and the cells were incubated under stimulated conditions. CGI-58 was not detected in immunoprecipitations of nontransduced cells, or when a nonimmune IgG was used for immunoprecipitations.

To determine whether CGI-58 is a substrate for PKA in intact cells or tissues, two types of experiments were conducted. Murine white adipose tissue was stimulated ex vivo with isoproterenol and IBMX to activate adenylyl cyclase, sustain elevated levels of cAMP, and, in turn, activate PKA. Immunoblots of 2D gels of the tissue lysates revealed three to four distinct spots detected by the CGI-58 antiserum that likely represent CGI-58 isoforms. Because phosphorylation adds negative charge, the isoelectric point of a phosphorylated protein is shifted to an acidic pH, as was observed in the PKA-stimulated tissue when compared with control tissue (**Fig. 4A**). Additionally, CGI-58 was immunoprecipitated from the stimulated and control tissues and immunoblotted with an anti-phospho-S/T PKA substrate antibody or CGI-58 antiserum (Fig. 4B). Only the stimulated tissue showed a positive signal for phosphorylation, while similar amounts of CGI-58 were detected in immunoprecipitates from both the stimulated and control samples. Finally, to confirm that Ser239 is the major site for phosphorylation of CGI-58 by PKA in intact cells, CGI-58 was immunoprecipitated from stimulated and control NIH3T3CARΔ cells expressing intact CGI-58 (WT) or S239A/S240A-mutated CGI-58 (Fig. 4C). As expected, only stimulated cells expressing intact CGI-58 showed detectable phosphorylation.

### PKA-mediated phosphorylation of Ser239 does not alter CGI-58 function in either coactivation of ATGL in vitro or turnover of triacylglycerol in NLSD fibroblasts

**Fig. 5.**
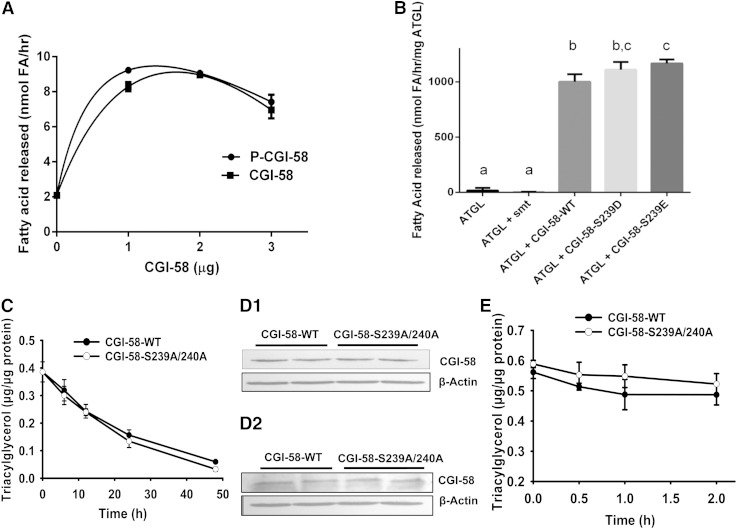
PKA-mediated phosphorylation of Ser239 does not alter the role of CGI-58 in coactivation of ATGL or turnover of triacylglycerol in NLSD fibroblasts. A: Purified recombinant CGI-58 was phosphorylated in vitro prior to incubation with Sf9 cell lysates containing recombinant ATGL and radioactive triolein emulsified with 3:1 phosphatidylcholine:phosphatidylinositol. Released fatty acids were solvent-extracted and quantified by scintillation counting. Phosphorylated and nonphosphorylated CGI-58 were equally effective in increasing the triacylglycerol hydrolytic activity of ATGL. Experiment is a representative experiment out of three, each with triplicate samples; data are mean ± standard deviation of triplicate samples. B: Recombinant WT or S239D- or S239E-mutated CGI-58, or extracts from *E. coli* expressing the control vector driving the production of a 6-His-Sumo protein (smt), were mixed with partially purified recombinant truncated ATGL (amino acids 1–288) and radiolabeled triacylglycerol substrate emulsified with phospholipids for determination of ATGL activity. WT CGI-58 and S239D- and S239E-mutated CGI-58 were equally effective in activating triacylglycerol hydrolytic activity of ATGL in vitro. Data are mean ± standard deviation of triplicate samples from one representative experiment out of two. Data were analyzed by ANOVA; data with the same letter subscript are not different from each other; data with different letters are different (*P* < 0.05). C: Adenoviral vectors were used to express either WT CGI-58 or S239A/S240A-mutated CGI-58 in NLSD fibroblasts. At the indicated times, lipids were solvent-extracted and the triacylglycerol content determined by an enzymatic assay. Triacylglycerol levels decreased at 6 h and all subsequent time points. Data are the mean ± standard error of the mean for triplicate samples from a representative experiment out of two. Data were analyzed by ANOVA; no significant differences were observed between WT CGI-58 and S239A/S240A CGI-58. D: Immunoblotting was used to determine the relative expression of intact (WT) CGI-58 and S239A/S240A-mutated CGI-58 at 24 h (D1) and 48 h (D2). E: Adenoviral vectors were used to express WT CGI-58 and S239A/S240A-mutated CGI-58 in NLSD fibroblasts. Six hours after the addition of adenoviruses, cells were incubated with forskolin and IBMX. Lipids were extracted and triacylglycerol levels determined. The addition of forskolin and IBMX to NLSD cells does not increase the rate of triacylglycerol turnover in the presence of either WT or mutated CGI-58. Data are mean ± standard error of the mean for triplicate samples. When error bars are not visible they are contained within the symbols.

To gain understanding of the function of CGI-58 phosphorylation, CGI-58-mediated coactivation of the triacylglycerol hydrolase activity of ATGL was assessed in vitro. Purified recombinant CGI-58 was incubated with PKA and nonradioactive ATP prior to incubation of phosphorylated and nonphosphorylated CGI-58 with Sf9 insect cell lysates containing recombinant mouse ATGL and an emulsified radioactive triacylglycerol substrate. Phosphorylated and nonphosphorylated CGI-58 showed equivalent capacity to activate the triacylglycerol hydrolase activity of ATGL over hydrolysis catalyzed by ATGL alone (**Fig. 5A**). Similar results were obtained when CGI-58 with S239D or S239E phosphomimetic mutations were used to activate a partially purified truncated variant (amino acids 1–288) of recombinant ATGL (Fig. 5B). The data indicate that the phosphorylation of CGI-58 neither facilitates nor impedes the coactivation of ATGL.

To test CGI-58 function in intact cells, adenoviral expression vectors were employed to drive the expression of either intact CGI-58 (WT) or S239A/S240A-mutated CGI-58 in cultured human NLSD fibroblasts that lack functional CGI-58 (10), and have perilipin 2-containing lipid droplets (not shown). Immunoprecipitations revealed that NLSD cells express ATGL (data not shown), yet are unable to turn over triacylglycerol normally in the absence of CGI-58 (27). Without ectopic expression of CGI-58, NLSD cells expressing β-galactosidase as a control protein show approximately 15-fold higher triacylglycerol levels relative to fibroblasts from control humans cultured in the same media (supplementary Fig. 1). By 6 h after transduction with adenoviruses for either the intact (WT) or mutated variant of CGI-58, the triacylglycerol content of NLSD cells decreased significantly (relative to untransduced cells; *t* = 0 h), and continued to decrease over 48 h (Fig. 5C). The rate of triacylglycerol turnover was similar for cells expressing WT and mutated CGI-58. Immunoblotting of cell lysates for CGI-58 revealed that 6 h is sufficient to detect ectopic CGI-58 (data not shown); levels of CGI-58 increased with longer time of protein expression. Moreover, equivalent levels of intact and mutated CGI-58 were detected (Fig. 5D). In summary, S239A/S240A-mutated CGI-58 was as effective as WT CGI-58 in facilitating the turnover of triacylglycerols in NLSD cells.

Initial experiments to test CGI-58 function in NLSD fibroblasts were conducted in the absence of compounds to activate PKA. We next investigated whether the activation of PKA would alter the rate of triacylglycerol turnover in NLSD cells expressing ectopic CGI-58. Adenoviruses driving the expression of either intact (WT) or S239A/S240A-mutated CGI-58 were added to NLSD cells 6 h prior to the addition of forskolin and IBMX. Measurement of the triacylglycerol content of cell lysates over the next 2 h revealed no increase in the rate of lipolysis due to the activation of PKA, and no difference in the rate of triacylglycerol turnover when comparing data from cells expressing WT CGI-58 to those from cells expressing S239A/S240A-mutated CGI-58 (Fig. 5E). Thus, PKA is not a major modulator of lipolysis in NLSD fibroblasts, and phosphorylation of Ser239 (or Ser240) is not required for the function of CGI-58 in facilitating triacylglycerol turnover in human skin fibroblasts. It is important to note that fibroblasts lack both perilipin 1 and hormone-sensitive lipase; hence, the major players in PKA-mediated lipolysis are absent in these cells.

### PKA-mediated phosphorylation of Ser239 alters the subcellular localization of CGI-58 in forskolin-treated cells expressing perilipin 1A

**Fig. 6.**
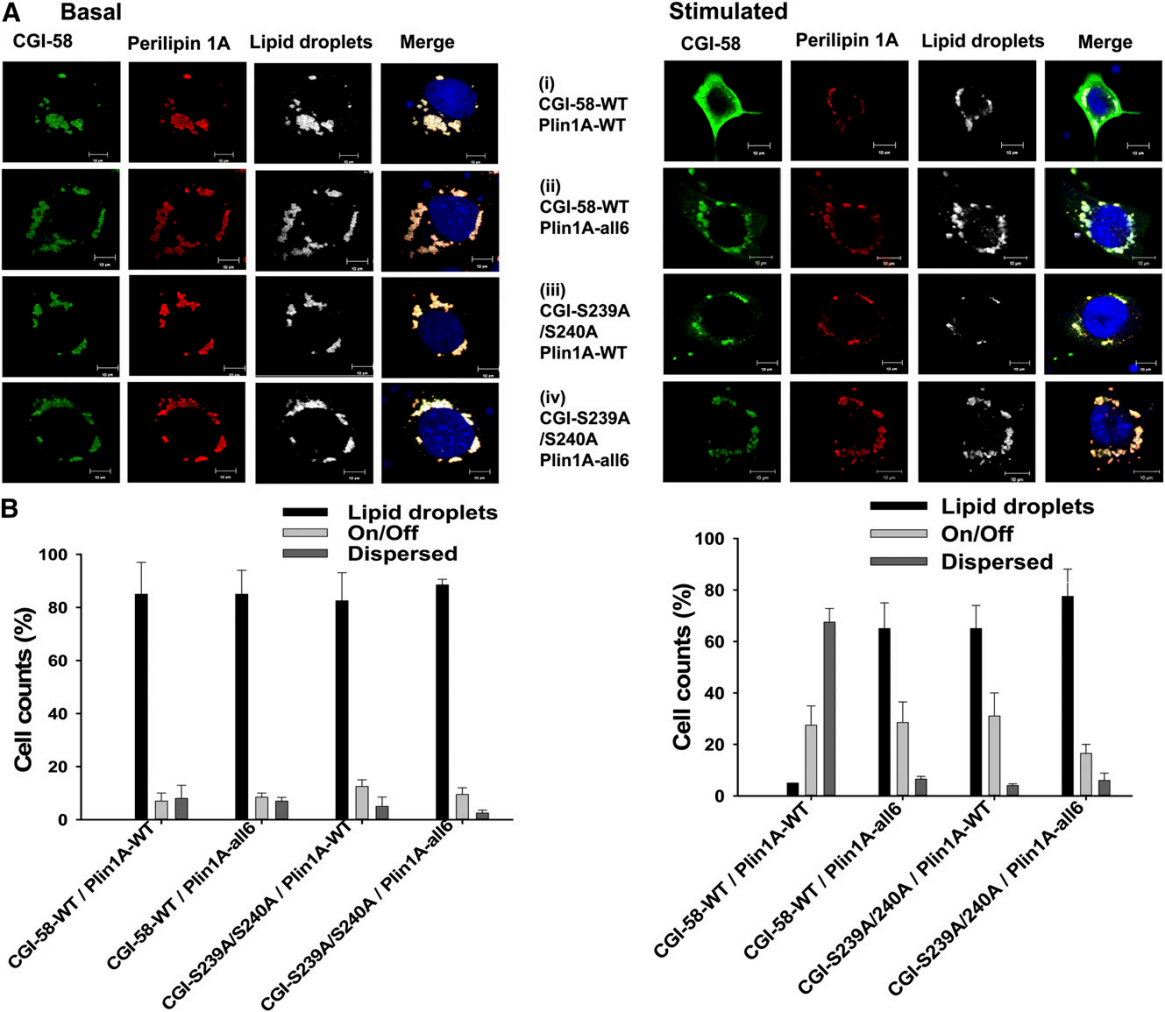
CGI-58 and S239/S240A-mutated CGI-58 localize equally well to lipid droplets coated with perilipin 1A, but mutation of serine residues in PKA consensus sequences of either CGI-58 or perilipin 1A reduces forskolin-induced dispersion of CGI-58 into the cytoplasm. A: Representative micrographs of Cos-7 cells expressing WT CGI-58 [CGI-58-WT (i, ii)] or S239A/S240A-mutated CGI-58 [CGI-S239A/S240A (iii, iv)] and WT perilipin 1A [Plin1A-WT (i, iii)] or perilipin 1A with mutations in six serine residues within PKA consensus sequences [Plin1A-all6 (ii, iv)] under basal (left side panels) or forskolin + IBMX stimulated conditions (right side panels). Cells were fixed and stained with antisera against perilipin and CGI-58, Hoechst 33422 for the detection of nuclei, and BODIPY 493/503 for the detection of lipid droplets. B: Averaged cell counts for two observers counting more than 50 cells per condition for both basal (left side) and forskolin + IBMX incubated (right side) cells. “Lipid droplets” designates signal for CGI-58 primarily on lipid droplets; “On/Off” designates signal for CGI-58 both on lipid droplets and diffuse throughout the cytoplasm; “Dispersed” designates signal for CGI-58 primarily diffuse throughout the cytoplasm. Experiment was repeated at least three times in each of two locations.

We next asked whether the PKA-mediated phosphorylation of CGI-58 affects subcellular localization of the protein. PKA controls lipolysis in adipocytes in part by modulating the subcellular localization of CGI-58 (8, 11, 37). Under basal conditions, CGI-58 binds to perilipin 1A on adipocyte lipid droplets, but when PKA is activated, CGI-58 disperses into the cytoplasm (8, 11). The PKA-mediated phosphorylation of carboxyl terminal serine residues of perilipin 1A has been shown to facilitate the dispersion of CGI-58 from lipid droplets (11); this event is necessary for CGI-58 to gain access to ATGL. We hypothesized that the PKA-mediated phosphorylation of CGI-58 might also contribute to the translocation of CGI-58 into the cytoplasm in lipolytically stimulated cells. Experiments were conducted in both cultured Cos-7 cells and NIH3T3CARΔ fibroblasts; the latter cells lack detectable endogenous CGI-58, as determined by immunoprecipitation followed by immunoblotting (Fig. 4C). Using adenoviral expression vectors, either intact CGI-58 (WT) or S239A/S240A-mutated CGI-58 was expressed with either intact (WT) perilipin 1A or a mutated variant of perilipin 1A lacking serine residues in six consensus PKA sites. Adenoviral titers were adjusted to drive equivalent levels of protein expression for both variants of perilipin 1A and both variants of CGI-58 (supplementary Fig. 2). For 12 h prior to starting the experiment, the cells were incubated with oleic acid to increase triacylglycerol synthesis and lipid droplet formation, and consequently, stabilize perilipin 1A (38). Cells were then incubated for 30 min with forskolin and IBMX, or under basal conditions, before fixation and immunostaining of the cells for perilipin and CGI-58. The data show that both intact (WT) and S239A/S240A-mutated CGI-58 localized to perilipin-coated lipid droplets under basal conditions, whether WT or mutated variants of perilipin 1A were expressed (**Fig. 6A, B**; **Table 3**). In contrast, the mutation of PKA-site serine residues in CGI-58 and perilipin 1A decreased the dispersion of CGI-58 into the cytoplasm following incubation of the cells with forskolin and IBMX, whether only one protein was mutated or both were mutated in combination. Thus, the PKA-mediated phosphorylation of both CGI-58 and perilipin 1A contribute to the dispersion of CGI-58 from perilipin 1A-coated lipid droplets.

**TABLE 3.** Protein colocalization analysis by determination of Manders coefficients

Expressed Proteins		Colocalization of CGI-58 with Perilipin 1A (M2)		Colocalization of CGI-58 with Lipid Droplets (M2)	
CGI-58	Perilipin 1A	Basal	Stimulated	Basal	Stimulated
WT	WT	0.86 ± 0.03	0.46 ± 0.03	0.86 ± 0.02	0.46 ± 0.03
WT	All6	0.98 ± 0.01	0.65 ± 0.03	0.96 ± 0.01	0.63 ± 0.03
239A/240A	WT	0.88 ± 0.02	0.65 ± 0.03	0.79 ± 0.03	0.62 ± 0.03
239A/240A	All6	0.83 ± 0.03	0.84 ± 0.04	0.80 ± 0.03	0.88 ± 0.03

Cos-7 cells expressing WT CGI-58 (Fig. 6Ai, ii; CGI-WT) or S239A/S240A-mutated CGI-58 (Fig. 6Aiii, iv; CGI-S239A/S240A), and WT perilipin 1A (Plin1A-WT) or perilipin 1A with mutations in six serine residues within PKA consensus sequences (Plin1A-all6) were incubated under basal or stimulated conditions before fixation and staining for CGI-58, perilipin 1, nuclei, and lipid droplets. Micrographs were analyzed automatically with the ImageJ JACoP plugin. Colocalization of CGI-58 with either perilipin 1A or lipid droplets was assessed by determination of Manders coefficients 2 (M2) (mean ± standard error of the mean). An M2 of 0.00 indicates no colocalization, while 1.00 indicates complete colocalization.

We conducted a similar experiment using cells expressing S239D CGI-58 and perilipin 1A. Under basal conditions, S239D CGI-58 localized efficiently to perilipin 1A-coated lipid droplets (supplementary Fig. 3), suggesting that the addition of negative charge to amino acid 239 of CGI-58 is insufficient to cause dispersion of CGI-58 from perilipin 1A-coated lipid droplets without the activation of PKA. When cells were stimulated with forskolin and IBMX, S239D CGI-58 efficiently dispersed into the cytoplasm (supplementary Fig. 3). These data suggest that negative charge at position 239 is important for CGI-58 dispersion when PKA has been activated.

## DISCUSSION

In this study, we employed a variety of approaches to demonstrate that CGI-58 is phosphorylated on Ser239 by PKA. The finding is supported by data from the phosphorylation of recombinant CGI-58 in vitro, immunoblotting of immunoprecipitated proteins from mammalian tissue and cells in which phosphorylation occurred in intact cells, and MS. All of our studies were conducted using mouse CGI-58; however, human CGI-58 is likely phosphorylated, given the complete conservation of the PKA consensus sequence in chordates.

In adipocytes, the PKA-mediated phosphorylation of key mediators of lipolysis is a crucial event in the initiation of lipolysis. Phosphorylation of hormone-sensitive lipase is required to trigger movement of the lipase from a diffuse cytoplasmic localization to the surfaces of lipid droplets (39–42), and to activate lipase activity in an as yet poorly understood mechanism (12, 43, 44). The phosphorylation of perilipin 1A is also required to enable hormone-sensitive lipase docking on lipid droplets through protein-protein interactions between the lipase and perilipin (12). Additionally, phosphorylation of perilipin 1A is required to release CGI-58 from its binding site on a carboxyl terminal sequence of perilipin 1A (8, 11); this redistribution of CGI-58 into the cytoplasm increases the interaction of CGI-58 with ATGL (11). We now demonstrate that PKA-mediated phosphorylation of CGI-58 also plays a role in the dispersion of CGI-58 from the perilipin scaffold, to enable CGI-58 interaction with and coactivation of ATGL. A previous study has demonstrated that perilipin 1A sequestration of CGI-58 prevents the interaction of CGI-58 and ATGL, reducing basal lipolysis; human truncation mutations in perilipin 1A that prevent CGI-58 sequestration lead to elevated rates of basal lipolysis (45). Thus, precise control of the subcellular localization of CGI-58 is an important component of the regulation of lipolysis in adipocytes.

Although the PKA-mediated phosphorylation of CGI-58 is an important step in the activation of lipolysis for cells in which perilipin 1A is the major lipid droplet-associated protein, it is likely less important for cells in which lipid droplets are coated with other members of the perilipin family. Perilipin 1A is an abundant lipid droplet protein in adipocytes from white and brown adipose tissue (46), is less abundant in steroidogenic cells of adrenal cortex, testes, and ovaries (47), and is largely absent from other cells and tissues under normal physiological conditions. In steroidogenic cells, hormonal signaling activates adenylyl cyclase, in turn, activating PKA, and initiating the lipolysis of stored cholesterol esters to provide substrate for steroid hormone synthesis (48). Although CGI-58 is expressed in steroidogenic cells, it has not been shown to play a role in lipolysis of cholesterol esters. Most other cells of the body, including human skin fibroblasts, have small lipid droplets coated with perilipin 2 (35), which does not appear to be phosphorylated by PKA. In these cells, it is unlikely that phosphorylation of CGI-58 plays a role in facilitating lipolysis. Consistent with this concept, the addition of forskolin and IBMX to cultured fibroblasts from an individual with NLSD did not accelerate lipolysis in the presence of ectopic CGI-58. Moreover, CGI-58 does not localize strongly to lipid droplets when perilipin 2 is the predominant lipid droplet-associated protein, but instead displays a diffuse localization pattern throughout the cytoplasm (8, 49). It is possible that PKA-mediated phosphorylation of CGI-58 plays a role in lipolysis in myocytes from cardiac and skeletal muscle; perilipin 5 is a major lipid droplet protein in these cells (50–52). CGI-58 localizes to lipid droplets by binding to carboxyl-terminal sequences of perilipin 5, but does not appear to interact with ATGL when bound to perilipin 5 (49). Recent experiments have suggested that PKA-mediated phosphorylation events increase lipolysis in cells expressing perilipin 5, and that perilipin 5 may be a substrate for PKA (53). Additional experimentation is required to determine whether the phosphorylation of CGI-58 facilitates lipolysis in cells expressing perilipin 5 through a dispersion mechanism similar to that observed in perilipin 1A expressing cells.

Although the phosphorylation of CGI-58 increases the availability of CGI-58 to bind to ATGL in adipocytes, it does not affect the coactivation mechanism itself. In vitro lipolysis assays suggest that phosphorylated CGI-58 activates ATGL as efficiently as nonphosphorylated protein. The mechanism by which CGI-58 increases ATGL-mediated hydrolysis of triacylglycerols is as yet unknown. Control of ATGL activity is complex; triacylglycerol hydrolase activity is inhibited by a small protein named G0S2 (54, 55), yet there does not appear to be competition between G0S2 and CGI-58 for access to ATGL. The mechanism of G0S2 inhibition of ATGL is as yet unknown, as is how a transition is made between G0S2 inhibition and CGI-58 activation of lipase activity.

We have shown that Ser239 of CGI-58 is phosphorylated by PKA in vitro, as well as in tissue samples and intact cultured cells. Interestingly, 2D electrophoresis revealed three to four isoforms of CGI-58, suggesting additional posttranslational modifications. Additional studies are needed to address this hypothesis.

## Supplementary Material

Supplemental Data
