# Supplementary material for: CGI-58/ABHD5 is phosphorylated on Ser239 by protein kinase A: control of subcellular localization
Source: J Lipid Res. 2015 Jan;56(1):109–21. doi: 10.1194/jlr.M055004 (PMC4274058; doi:10.1194/jlr.M055004)
Supplement: Supplemental Data [file supp_56_1_109__index.html]

CGI-58/ABHD5 is phosphorylated on Ser-239 by protein kinase A: Control of subcellular localization — CGI-58/ABHD5 is phosphorylated on Ser239 by protein kinase A: control of subcellular localization — Supplemental Data 

# CGI-58/ABHD5 is phosphorylated on Ser239 by protein kinase A: control of subcellular localization

## Supplemental Data

**Files in this Data Supplement:**

- Supplemental Figures.pdf - Supplemental Figures
