# Supplementary material for: CGI-58/ABHD5 is phosphorylated on Ser239 by protein kinase A: control of subcellular localization
Source: J Lipid Res. 2015 Jan;56(1):109–21. doi: 10.1194/jlr.M055004 (PMC4274058; doi:10.1194/jlr.M055004)
Supplement: Supplemental Data [file supp_M055004_jlr.M055004-1.pdf]

Supplemental Data

CGI-58/ABHD5 is phosphorylated on Ser-239 by protein kinase A: Control of subcellular localization

Anita Sahu-Osen, Gabriela Montero-Moran, Matthias Schittmayer, Katarina Fritz, Anna Dinh, Yu-Fang Chang, Derek McMahon, Andras Boeszoermenyi, Irina Cornaciu, Deanna Russell, Monika Oberer, George M. Carman, Ruth Birner-Gruenberger, and Dawn L. Brasaemle

Supplemental Figures

Supplemental Figure 1. NLSD cells store excessive levels of triacylglycerol that can be alleviated by CGI-58 expression. NLSD cells were transduced with adenovirus to drive the expression of WT CGI-58, S239A/S240A mutated CGI-58, or β-galactosidase (control protein). Cells were harvested 48 hours after transduction and triacylglycerol levels were determined and compared to that of control human fibroblasts. NLSD cells expressing β-galactosidase stored 15-fold more triacylglycerol than control cells. The expression of both WT and mutated CGI-58 reduced levels of stored triacylglycerol.

Supplemental Figure 2. Titers of adenoviral vectors were adjusted for equivalent protein expression. Cos7 cells were transduced with adenoviral vectors to drive the expression of WT perilipin 1A (Plin1A), mutated Plin1A (Plin1A-all 6), WT CGI-58 and mutated CGI-58 (CGI-58-S239A/S240A); two vectors were simultaneously transduced into cells. Cells were harvested 48 hours after transduction and cell lysates were prepared for SDS-PAGE and immunoblotting for ectopic Plin1 (A) and CGI-58 (B) and endogenous actin (A and B) as a control for protein load.

Supplemental Figure 3. S239D mutated CGI-58 localizes to lipid droplets coated with perilipin 1A under basal conditions and disperses into the cytoplasm following the treatment of cells with forskolin and IBMX. Cos7 cells were transduced with adenovirus for the expression of WT perilipin 1A (*Plin1A-WT; i, iii*) or mutated perilipin 1A (*Plin1A-all6; ii, iv*) 12 hours before transfection with WT CGI-58 (*CGI-58-WT; i, ii*) or S239D CGI-58 (*CGI-58-S239D; iii, iv*) in the pcDNA™4/HisMax vector (Life Technologies) using Lipofectamine® 3000 (Life Technologies) for 12 hours. At 24 hours after transduction with adenovirus, cells were moved to 24-well glass-bottomed plates; at 36 hours after transduction, cells were lipid-loaded with 200 µM oleic acid complexed to fatty acid-free albumin (4:1 molar ratio) for 12-16 hours. At 52 hours after transduction with adenovirus, media on half of the wells were changed to stimulation medium (10 µM forskolin, 0.5 mM IBMX, 2% fatty acid-free BSA in DMEM; *right side*); the remaining wells received basal medium (2% fatty acid-free BSA in DMEM with

DMSO vehicle; *left side*). Cells were incubated at 37°C for 30 minutes prior to fixation and staining for CGI-58 (*green*), perilipin 1A (*red*), lipid droplets (Bodipy 493/503; *white*) and nuclei (Hoechst 33422; *blue*). **A)** Representative micrographs; **B)** Averaged cell counts for 2 observers counting more than 50 cells per condition for both basal (*left side*) and forskolin + IBMX incubated (*right side*) cells. "*Lipid Droplets*" designates signal for CGI-58 primarily on lipid droplets; "*On/Off*" designates signal for CGI-58 both on lipid droplets and diffuse throughout the cytoplasm; "*Dispersed*" designates signal for CGI-58 primarily diffuse throughout the cytoplasm.

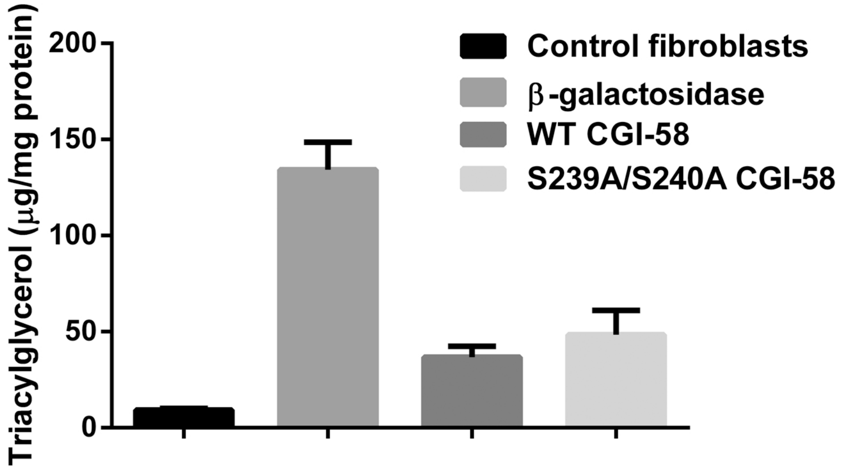

Supplemental Figure 1

A

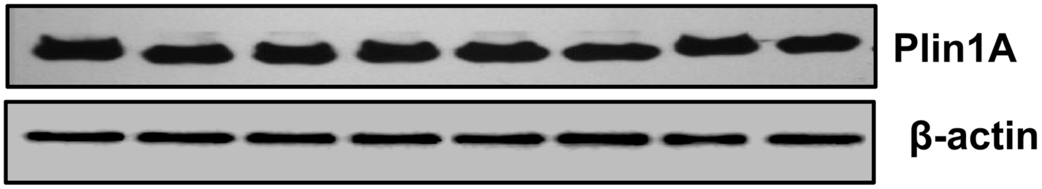

2 µl	2 µl			1.5 µl	1.5 µl			Plin1A-WT
		1.5 µl	1.5 µl			1 µl	1 µl	Plin1A-all6
15 µl		15 µl		10 µl		10 µl		CGI-58-WT
	20 µl		20 µl		16 µl		16 µl	CGI-239A/240A

B

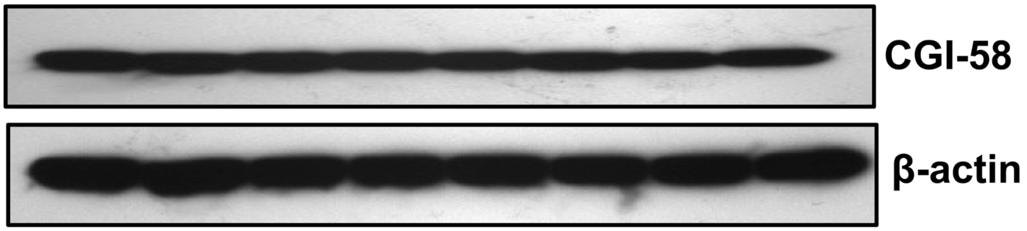

15 µl	15 µl			10 µl	10 µl			CGI-58-WT
		20 µl	20 µl			16 µl	16 µl	CGI-239A/240A
2 µl		2 µl		1.5 µl		1.5 µl		Plin1A-WT
	1.5 µl		1.5 µl		1 µl		1 µl	Plin1A-all6

Supplemental Figure 2.

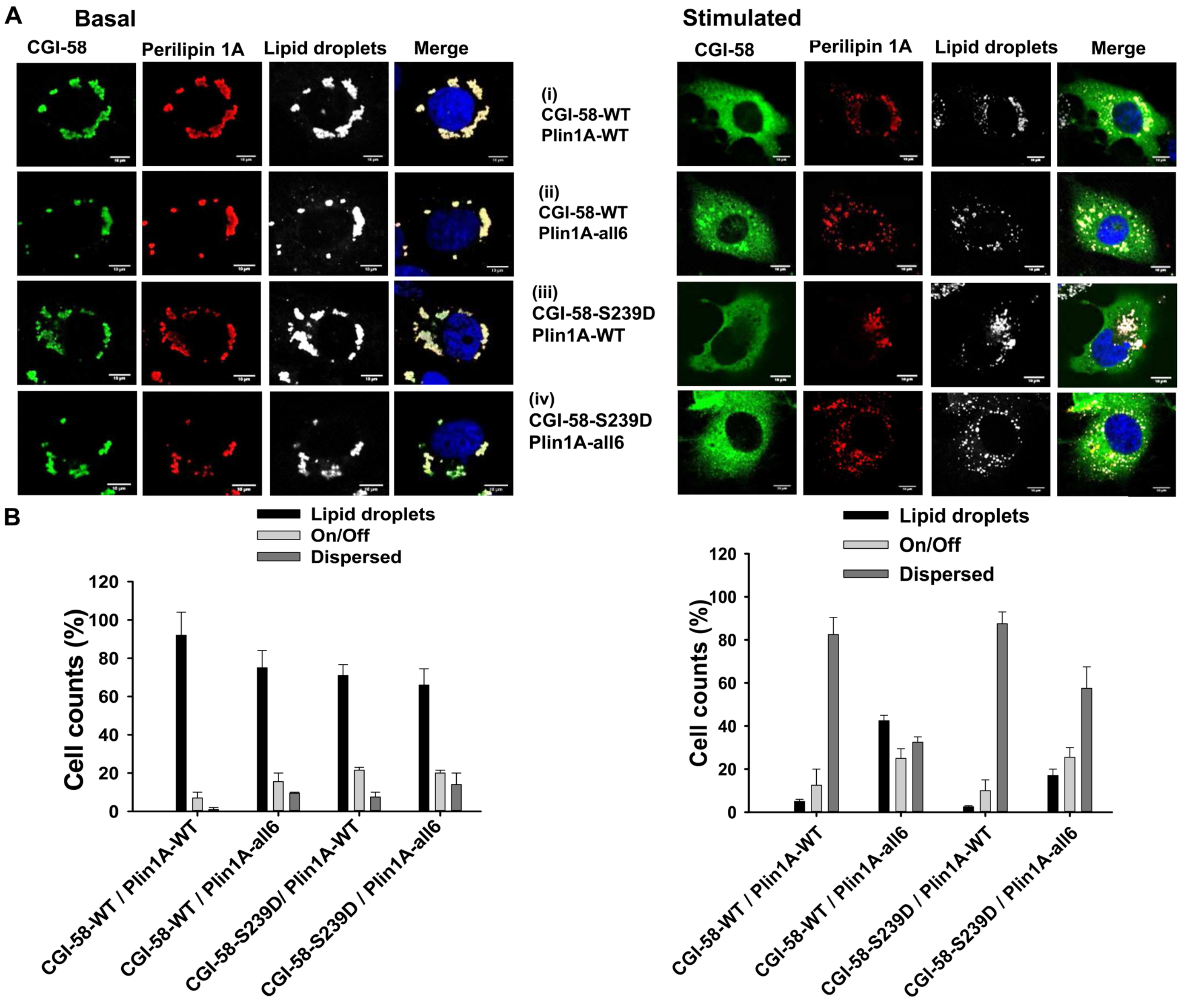

Supplemental Figure 3
